# Beet Stalks and Leaves (*Beta vulgaris* L.) Protect Against High-Fat Diet-Induced Oxidative Damage in the Liver in Mice

**DOI:** 10.3390/nu10070872

**Published:** 2018-07-05

**Authors:** Isabela M. Lorizola, Cibele P. B. Furlan, Mariana Portovedo, Marciane Milanski, Patrícia B. Botelho, Rosângela M. N. Bezerra, Beatriz R. Sumere, Maurício A. Rostagno, Caroline D. Capitani

**Affiliations:** 1 School of Applied Sciences, University of Campinas, Limeira, SP 13484-350, Brazil; isabela_lorizola@hotmail.com (I.M.L.); mariportovedo@gmail.com (M.P.); marciane.milanski@fca.unicamp.br (M.M.); rosangela.bezerra@fca.unicamp.br (R.M.N.B.); beatriz.sumere@fca.unicamp.br (B.R.S.); mauricio.rostagno@gmail.com (M.A.R.); 2 Faculty of Food Engineering—FEA, Department of Food and Nutrition—DEPAN, University of Campinas, Campinas, SP 13484-350, Brazil; cibelefurlan07@gmail.com; 3 Faculty of Nutrition—FANUT, Federal University of Goias—UFG, Goiânia, GO 74.605-080, Brazil; patriciaborges.nutri@gmail.com

**Keywords:** functional foods, phenolic compounds, antioxidant enzymes

## Abstract

Some flavonoids identified in beet stalks can help the antioxidant endogenous defenses during a chronic inflammation process. The current study investigates the effect of polyphenols present in beet stalks and leaves on liver oxidative damage in mice fed a high-fat diet (HF). The control (CT) or HF diet groups were supplemented with dehydrated beet stalks and leaves (SL) or beet stalk and leaf ethanolic extract (EX). In terms of Vitexin-rhaminoside equivalents (VRE), EX groups received ~5.91 mg of VRE·100 g^−1^ diet, while the SL groups received ~3.07 mg VRE·100 g^−1^ diet. After 8 weeks, we evaluated fasting blood glucose; cholesterol, hepatic Malondialdehyde (MDA) levels and hepatic Glutathione (GSH), Glutathione peroxidase (GPx), Glutathione reductase (GR) and Superoxide dismutase (SOD) activity. Dehydrated beet stalks and leaves (HFSL) attenuated the deleterious effects of a HF diet on lipid metabolism, reduced fasting blood glucose levels, ameliorated cholesterol levels and reduced GPx and GR activities (*p* < 0.05) compared to the HF group. However; the addition of ethanolic extract from beet stalks and leaves was unable (*p* > 0.05) to prevent the liver damage caused by HF diet in mice. The presence of flavonoids, such as Vitexin derivatives in beet stalks and leaves can help the liver damage induced by HF diet.

## 1. Introduction

Obesity is associated with low-grade chronic inflammation that is strongly associated with oxidative stress [1,2]. The prevention or retardation of the oxidative process that is generated by chronic systemic inflammation is mediated by endogenous and exogenous antioxidants. Supplementation with exogenous antioxidants through diet or via supplements may help reduce oxidative stress and the risk of obesity-related complications [3,4]. Exogenous antioxidant compounds, which also have anti-inflammatory effects, such as vitamins, minerals and polyphenols, are largely present in vegetables, fruits, wine and teas [5,6,7], for example. Red beetroot (*Beta vulgaris* L.), is a vegetable that is traditionally consumed throughout the world, and is a source of antioxidant compounds [8]. The presence of different phenolic compounds in beetroots confers antioxidant properties to this vegetable [9] and recent studies have highlighted the role of nitrate derived from beetroots [10,11,12] in reducing blood pressure [13,14], and ameliorating type 2 diabetes mellitus [15].

Moreover, a positive effect of beetroot and of some of their derivatives on oxidative stress and inflammation has been demonstrated by Kujawska et al. (2009) [16] and Vulić et al. (2014) [17]. Koubaier et al. (2014) identified phenolic acids (gallic, vanillic, chlorogenic, ferulic, caffeic, syringic) and flavonoids (myricetin, quercetin, rutin, and kampferol) in beet stems [18]. Some flavonoids glycosides derived from Apigenin, namely Vitexin, have been associated with the beneficial effects of these plants on chronic inflammation, due to the inhibition of α-glucosidase activity [19,20] and increased low-density lipoprotein (LDL) resistance to oxidation [21,22,23]. According to Kannan and Jain (2000), inclusion of beet stalks as part of a healthy diet could serve as a strategy to enhance endogenous antioxidant defenses, helping to protect cellular components from oxidative damage [24]. In 2015, Clifford et al. concluded that beetroot supplementation can have positive effects on different aspects of health and disease, and may therefore represent an economical, practical and important product from the point of view of natural dietary intervention [25]. However, despite evidence of the benefits of beetroot, few studies have investigated the effects of the less-consumed parts of the beet, such as its stalks and leaves. These parts of the beet could be consumed in order to reduce food waste and to increase the nutritional value of meals [26]. Thus, the aim of the present study was to evaluate the effect of beetroot stalks and leaves on oxidative stress, using an experimental model of high-fat diet-induced obesity.

## 2. Materials and Methods

Organic beetroot stalks and leaves (*Beta vulgaris* L.) were obtained from an organic vegetable garden in Limeira city, São Paulo state, Brazil, during May and April 2015.

### 2.1. Drying Process

Beet stalks and leaves were sanitized, and excess moisture was removed at room temperature. Stalks/leaves were laid on aluminum trays and then placed in a pre-heated kiln (Clarice Brand, Queen, Pinhalzinho, Brazil) at 180 °C for 5 min. Previous analyses showed that the best conditions for drying the samples were at 180 °C for 30 min. The samples were homogenized and stored in hermetically-sealed containers at −80 °C until the time of analysis.

### 2.2. Beet Stalk and Leaf Extract Preparation

Dried beet leaves and stalks (2.5 ± 0.1 g) were placed in a conical centrifuge tube with 20 mL of ethanol (p.a.), before shaking (tube shaker, Phoenix luferco AP56) for 5 min, followed by centrifugation (5810 R Centrifuge-Germany) at 4000 rpm for 15 min at room temperature (25 ± 1 °C). After centrifugation, the supernatant was filtered through Whatman n° 3 filter paper, and an additional 20 mL of ethanol (p.a.) were added to the residue before repeating the process under the same conditions. The extracts obtained from the two sequential sample extractions were mixed and the volume was made up to 50 mL with ethanol (p.a.) in a volumetric flask. The extracts were stored at −80 °C in amber glass vials wrapped with aluminum foil until the time of analysis. The same ethanolic extract was used to supplement the diet (EX) and was also analyzed for total and individual phenolic compound concentration. To determine total and individual phenolic compound concentration, the ethanolic extract was filtered through syringe filters (nylon, 25 mm, 0.22 μm, Analytica, Barueri, Brazil) before ultra-high pressure liquid chromatography-tandem mass spectrometer (UHPLC-MS/MS) analysis.

### 2.3. Identification of the Compounds Present in the Extracts by UHPLC-MS/MS

The compounds present in the extract were identified using an UHPLC–MS/MS 8040 (Shimadzu, Kyoto, Japan) instrument consisting of a liquid chromatography system coupled to a triple quadrupole mass spectrometer, equipped with an electrospray ionization (ESI) source. Chromatographic separation was performed on a 2.6 μm, 3.0 mm i.d., 100 mm C18 Kinetexcolumn (Phenomenex, Torrance, CA, USA) using a binary mobile phase. Solvent A was water and solvent B was acidified acetonitrile (0.1% formic acid). The elution gradient used at 40 °C was as follows: 0 min: 98%A; 5 min: 98%A; 15 min: 85%A; 20 min: 80%A; 25 min: 65%A; 30 min: 20%A; 34 min: 20%A; 35 min: 98% A, at a flow rate of 0.3 mL·min^−1^. The autosampler temperature was maintained at 10 °C and the injection volume was 10 μL. The ESI source parameters were as follows: capillary voltage, −3.5 kV; heat block temperature, 500 °C; desolvation line temperature, 250 °C; drying gas flow (N2), 10 L·min^−1^; nebulizing gas flow (N2), 1.5 L·min^−1^; collision induced dissociation gas pressure (Ar), 224 kPa. For each compound, ESI(-)-MS/MS data were first collected for the identification of deprotonated molecules [M−H] and two of the most selective product ions were chosen for the MRM transitions using a dwell time of 20 ms. Data were acquired and processed with Labsolution software (version 5.53 SP2, Shimadzu). The recorded masses were processed throughout the chromatogram during the time interval of the peaks present at different wavelengths of detection (260, 290, 335, 360, 484 nm). The extract was then reinjected, the detected masses were examined for fragmentation using different capillary voltages and the detected fragments were recorded.

### 2.4. Determination of the Concentration of Phenolic Compounds by HPLC

The compounds present in the beet leaf and stalk extracts were analyzed by high performance liquid chromatography (HPLC) using the EXTRACT-US analysis system (FAPESP 2013/04304-4, patent pending), consisting of an HPLC pump (PU2080—Jasco, Tokyo, Japan), an HPLC ternary gradient unit (LG 2080-2—Jasco, Tokyo, Japan), a three line degasser (DG 2080-55, Jasco), a UV-vis detector (UV-7075, Jasco) and five automatic 2-way 10-port valves (Waters Corporation, Milford MA, USA). The compounds were separated by an adaptation of the method developed by Rostagno and coworkers [27] using a Fused-core type column (Kinetex C18, 2.6 μm, 100 A, 100 × 4.6 mm, Phenomenex, Torrance, CA, USA), which was maintained at room temperature. The mobile phase consisted of deionized water with 1% *v*/*v* phosphoric acid (solvent A) and acetonitrile with 1% *v*/*v* phosphoric acid (solvent B). The gradient profile was: 2 min, 88% A; 4 min, 80% A; 6 min, 70% A; 8 min 40% A; 10 min, 20% A; 13 min, 20% A and 14 min, 95% A. The equilibration time between runs was 3 min. The flow-rate was 1.2 mL·min^−1^ and injection volume was 5 μL. Peaks were recorded and integrated at 320 nm. The software for the control of the system was developed by Kalatec (Campinas, Brazil). The ChromNav software from Jasco was used for data acquisition and processing. The Vitexin-2-*O*-Rhamnoside compound was identified by comparing the retention times of the peak obtained in the analysis of the extracts to the peak obtained from the analysis of the authentic standard. The Vitexin-2-*O*-Rhamnoside standard solution (100 mg·L^−1^) was diluted in a mixture of methanol and water (90:10 *v*/*v*) to prepare the calibration curve (6 points: 100, 50, 25, 2.5, 1, 0.5 ppm). The calibration curve of the compound was prepared by plotting concentration versus area. All compounds present in the samples were expressed as vitexin 2-rhamnoside equivalents (VRE) and the analysis was performed in duplicate.

### 2.5. Chemical Composition

The moisture content of dried stalks and leaves was determined by vacuum oven drying at 100 °C up to a constant sample weight. The content of fixed mineral residue was analyzed by incinerating the sample on a hot plate and drying in a muffle at 550 °C until constant weight was obtained. Proteins were determined by the micro-Kjeldahl method [28] using a factor of 6.25 for conversion of total nitrogen. Lipids were extracted as described by Blight and Dyer (1959) [29]. The dietary fiber content was determined by the enzymatic-gravimetric method of Prosky et al. (1988) [30]. The carbohydrate content was calculated by the difference and the energy value was calculated by multiplying the protein and carbohydrate contents by 4 and the lipid content by 9 [28]. All analyses were performed in triplicate.

### 2.6. Experimental Protocol

Twenty-one-day-old Swiss male mice were obtained from the Center of Bioethics of UNICAMP. The experimental protocol of the study was approved by the Research Ethics Committee of UNICAMP (number ID 3206-1). The animals were allowed to acclimatize for 3 weeks before the beginning of the experiment and were then divided randomly into two groups: the control group, which was fed on a standard diet, and the experimental group, which was fed on a high-fat diet (Table 1). The ingredient compositions of the diets are described in Table 2.

**Figure 1:**
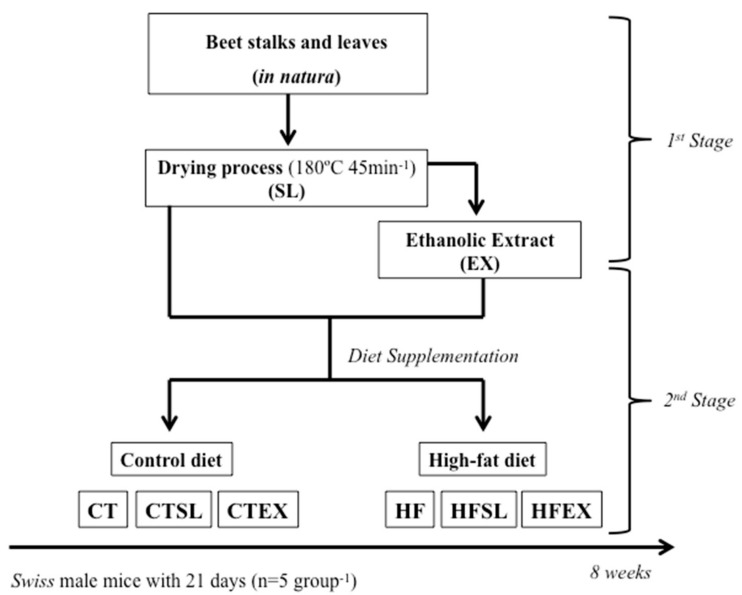
Experimental protocol. Stalks and leaves (SL); ethanolic extract (EX); the control (CT); standard diet with dried stalks and leaf (0.5% of dried stalks and leaves) (CTSL); : standard diet with stalk and leaf extract (190 mL of extract) (CTEX); high-fat diet (60% lipids) (HF); high-fat diet with dried stalks and leaf (60% lipids and 0.5% dehydrated beet stalks and leaves) (HFSL); high-fat diet with stalk and leaf extract (60% lipids and 19 mL of ethanolic extract) (HFEX).

Control animals (standard diet) were divided into three subgroups: (a) (CT) control group (*n* = 5); (b) (CTEX) control group fed on a diet containing the ethanol extract of dried beet stalks and leaves (*n* = 5); and (c) (CTSL) control group fed on a diet containing dried beet stalks and leaves (*n* = 5). The animals receiving the high-fat diet were also divided into three subgroups: (a) (HF) high-fat control group (*n* = 5); (b) (HFEX) high-fat group fed on a diet containing an extract of dried beet stalks and leaves (*n* = 5); and (c) (HFSL) high-fat group fed on a diet containing dried beet stalks and leaves (*n* = 5). Dehydrated beet stalks and leaves were mixed directly in the diet (0.5%; CTSL and HFSL). The ethanolic extract of beet stalks and leaves was added directly into the diet (CTEX and HFEX) and the total volume (19 mL·100 g^−1^) added in the diet (adjusted to increase the total phenolic compounds by two fold) was expressed as Vitexin-2-*O*-Rhamnoside and related compounds (VRE) 100 g^−1^ diet, compared to the phenolic concentration of the SL diets. Therefore, the group that was supplemented with dehydrated beet stalks and leaves (CTFL and HFSL) received 3.07 mg VRE·100 g^−1^ of diet and the animals supplemented with the ethanolic extract of beet stalks and leaves received 5.91 mg VRE·100 g^−1^ of diet. All groups were treated for 8 weeks (Figure 1).

The mice were housed in plastic cages (5 animals.cage^−1^) at constant temperature (22 ± 2 °C) and relative humidity (55 ± 10%), with a 12-h light–dark cycle. Food and water were available *ad libitum*. Animal weight and food intake were measured on an analytical scale (Mark Mark 500, Bel Engeenering, Italy), once a week during the 8 weeks of the experiment. At the end of the experiment, fasting glucose was measured with a glucose monitor (Glycometer, Bayer, Leverkusen, Germany) and the animals were anesthetized with sodium ketamine (0.1 g·kg^−1^), diazepam (5 mg·kg^−1^) and xylazine (3 mg·kg^−1^). After loss of paw and corneal reflexes, the animals were decapitated.

### 2.7. Tissue and Serum Sample Preparation

After the mice were sacrificed (*n* = 5 animals per group), the liver was removed, weighed and stored at −80 °C until further analysis. A liver homogenate was prepared with 0.1 M sodium phosphate buffer, pH 7.4. Blood samples were obtained by decapitation, and serum was separated by centrifugation at 3500 rpm for 30 min at room temperature (25 ± 1 °C) (Centrifuge 5417R—Eppendorf.). Total cholesterol (TC) was determined using a colorimetric method (Laborlab Kit number 1770080, São Paulo, Brazil). A standard solution concentration was diluted in 0.9% NaCl (ranging from 6.66 to 200 mg·dL^−1^) and used for the standard curve as a protocol procedure. Samples and the standard curve were read at 505 ηm with an automated biochemistry analyzer (Hitachi Ltd., Tokyo, Japan). Results are expressed as mg·dL^−1^. The analysis was performed in triplicate.

### 2.8. Malondialdehyde Concentration (MDA) Measurement

The MDA levels in plasma or tissue is an indicator of lipid peroxidation of cell membranes caused by chronic inflammation, for example. So, the hepatic MDA concentration was determined by high performance liquid chromatography (Agilent Technologies 1200 series—Santa Clara, CA, USA) following the protocol of Macedo et al. (2013) [31]. The MDA-TBA conjugate (20 μL) was injected into a Phenomenex C18 reverse phase analytical column (4.6 × 250 mm, 5 mm Phenomenex, Torrance, CA, USA) with an LC8-D8 pre-column (Phenomenex AJ0-1287, Torrance, CA, USA) and quantified fluorometrically at 515 nm excitation and 543 nm emission. The flow rate used was 1 mL·min^−1^, with a mobile phase consisting of 60% 10 mM potassium phosphate buffer, pH 7.0, and 40% methanol. A calibration curve was prepared using tetraethoxy propane (TEP) at increasing concentrations (0.5 to 15 μmol). For each sample employed for HPLC, a blank with milli-Q water and TEP (external control) at the concentration of 5 μmol was used. The analysis was performed in triplicate (*n* = 5 animals per group).

### 2.9. Antioxidant Enzyme Activity Measurement

Endogenous antioxidant enzyme activity is usually used as an indirect measure of increased generation of reactive oxygen species (ROS). Hence, liver homogenate samples (*n* = 5 animals per group) were used to determine glutathione peroxidase activity (GPx), total glutathione activity (GSH), glutathione reductase activity (GR) and superoxide dismutase activity (SOD). The liver protein concentration was determined by the Bradford method (1976) [32]. Antioxidant enzymes activities were expressed as mg·protein^−1^ of sample. All analyses were performed in triplicate, according to the methods described:

#### 2.9.1. Glutathione Peroxidase (GPx)

Liver GPx activity was determined by the method of Flohe and Gunzler (1984) with some adaptations, according to Silva et al. (2013) [33]. The reaction involved the oxidation of glutathione by hydrogen peroxide. Absorbance was measured with a microplate reader (Synergy-BIOTEK, Winooski, VT, USA) at 365 ηm and was continuously monitored over 10 min at 25 °C; enzyme activity was expressed as ηmol NADPH consumed.minute.mg protein^−1^.

#### 2.9.2. Total Glutathione (GSH)

Total glutathione activity was measured by performing a reaction with DTNB (20 mg in 5 mL methanol) in the liver and a standard curve was constructed with GSH at concentrations ranging from 0 to 500 ηmol·mL^−1^. The reaction involved 2 steps and was accompanied with a microplate reader (Synergy-BIOTEK, Winooski, VT, USA) at 412 ηm with 15 min interval between readings [33].

#### 2.9.3. Glutathione Reductase (GR)

Liver GR activity was determined by the method of Carlberg and Mannervick (1985), with adaptations according to Silva et al. (2013) [33], employing the reduction of oxidized glutathione by a reaction with NADPH. Absorbance was measured with a microplate reader (Synergy-BIOTEK, Winooski, VT, USA) at 340 ηm and was continuously monitored over 10 min at 25 °C; results were expressed as GR ηmol NADPH consumed.min.mg protein^−1^.

#### 2.9.4. Superoxide Dismutase (SOD)

Liver SOD activity was determined by the method of Winterbourn et al. (1995) with some adaptations [33]. Absorbance was measured with a microplate reader (Synergy-BIOTEK, Winooski, VT, USA) at 560 ηm and was continuously monitored every minute for 10 min at 25 °C. Activity was expressed as SOD U·mg protein^−1^.

### 2.10. Statistical Analysis

Data are presented as means ± SEM. After applying the normality test (Shapiro-Wilk test), two-way multiple comparison analysis of variance (ANOVA two-way) was used to compare the means obtained within each group and the differences between the means were assessed using Tukey’s test. Statistical significance was considered at *p* < 0.05. All statistical analyses were performed, and graphs were prepared using the GraphPad Prism software version 5.00 (Trial).

## 3. Results

### 3.1. Composition of Beet Stalks and Leaves

The centesimal composition of beet stalks and leaves after conventional oven drying is presented in Table 3.

### 3.2. Concentration of Phenolic Compounds and Phenolic Compound Characterization

**Figure 2:**
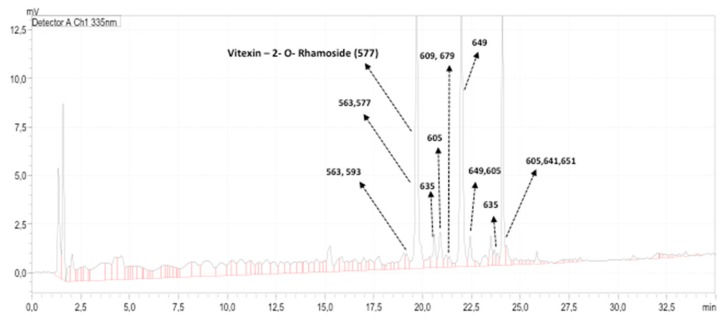
Ultra-high pressure liquid chromatography—tandem mass spectrometer (UHPLC-MS/MS) fingerprinting of the leaf extract obtained at 335 ηm and the ions (*m*/*z*) of the compounds detected.

UHPLC-MS/MS analysis revealed the presence of several compounds in the ethanolic extract of the beet stalks and leaves. The chromatograms obtained at 260, 290, 335, 360 and 484 nm revealed the presence of 20 peaks with masses ranging from values as low as 117 *m*/*z* to values as high as 1127 *m*/*z* (Table 4). The chromatogram obtained at 335 ηm (where most peaks were detected) is shown in Figure 2.

**Figure 3:**
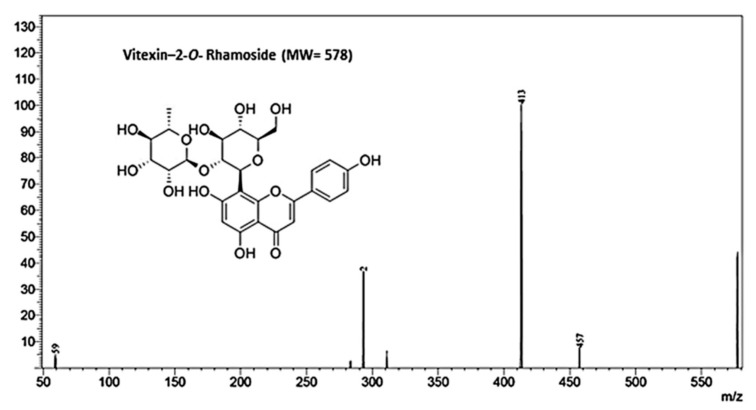
MS/MS fingerprinting of Vitexin-2-*O*-Rhamnoside, the main compound present in the ethanolic extract of beet stalks and leaves.

Based on the MS/MS spectra of the peak with the largest area, consisting of fragments (*m*/*z*) 457, 413 and 293 (Figure 3), the main compound identified in the extracts was Vitexin-2-*O*-Rhamnoside (MW 578), with a retention time of approximately 19.7 min (Figure 2). Comparison of the retention time and coelution of the authentic standard with the extract confirmed the identity of the peak. The concentration of Vitexin-2-*O*-Rhamnoside was 153.53 ± 3.5 mg·L^−1^, representing approximately 42% of the compounds present in the extract.

The other major compounds present in the extract presented a MW ranging from 564 to 652, and their ionization revealed the presence of the ion (*m*/*z*) 293, suggesting a relationship to Vitexin-2-*O*-Rhamnoside. Thus, it was possible to express the concentration of these compounds as Vitexin-2-*O*-Rhamnoside equivalents (VRE) to provide an estimate of their concentration in the extract. A total of 13 peaks were integrated at 320 nm in the chromatogram of the extract. The total concentration of the compounds present was 370.12 ± 6.2 mg VRE·L^−1^ and the mean concentration was 36.32 ± 47.12 mg VRE·L^−1^ extract.

However, it is important to point out that in terms of concentration, two other major peaks (RT~21.7–22.2 min and RT~23.96–24.24 min) were observed, in addition to Vitexin-2-*O*-Rhamnoside. The peak eluting at 21.7–22.2 min indicated a compound of MW 606 with main fragmentation ions of (*m*/*z*) 545, 455 and 293. The peak eluting at RT~23.96–24.24 min was a mixture of three compounds with MW 606, 642 and 652, all showing a spectrum similar to that of the compound eluting at 21.7–22.2 min, suggesting that they are closely related. Their concentrations in the extract were 67.04 ± 2.5 mg·L^−1^ and 65.93 ± 4.7 mg·L^−1^, respectively. In terms of relative concentration, these compounds represented approximately 18.1% and 17.8% of all the compounds in the extract, respectively. The concentration of Vitexin-2-*O*-Rhamnoside and these other two main compounds represented approximately 77.41% of all compounds present in the extract.

Therefore, when 0.5% dried stalks and beet leaves were added to the diets (CTSL and HFSL), the diets were found to contain approximately 3.07 mg VRE·100 g^−1^ diet with high amounts of Vitexin-2-*O*-Rhamnoside and related compounds. For the diets of the CTEX and HFEX groups, 19 mL extract per 100g feed was added and, thus, these diets contained 5.91 mg VRE·100 g^−1^ feed with high amounts of Vitexin-2-*O*-Rhamnoside and related compounds. Results also suggest that the leaf extract did not contain significant amounts of betalains or anthocyanins, as no peaks were detected at 484 nm.

### 3.3. The Effect of Beet Stalks and Leaves on Weight Gain, Blood glucose levels and Cholesterol Profile

**Figure 4:**
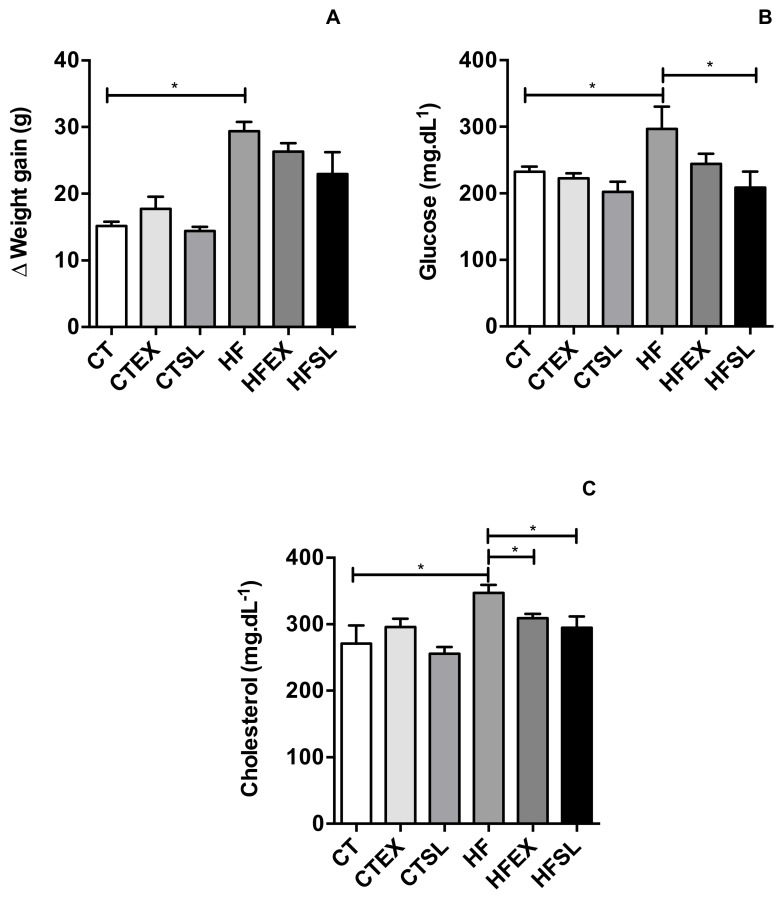
Animals weight gain, obtained by the difference between the final weight (9 weeks) and the initial weight of the animals in each group (**A**). Fasting blood glucose (mg·dL^−1^) of the animals at the end of the experiment (9 weeks) (**B**) Concentration of total cholesterol (mg·dL^−1^) in the plasma of the animals at the end of the experiment (9 weeks) (**C**) (*n* = 5 animals per group). CT: control group; CTEX: control group supplemented with beet stalks and leaves ethanolic extract; CTSL: control group supplemented with dehydrated beet stalks and leaves; HF: high-fat diet; HFEX: high-fat diet supplemented with beet stalks and leaves ethanolic extract; HFSL: high-fat diet supplemented with stalks and dried beet leaves. * Statistically significant difference (*p* < 0.05).

Feed intake did not differ (*p* > 0.05) between groups. The mean diet consumption was 4.69 g·day·animal^−1^ and this intake was equivalent to the average daily dose of vitexin rhamnoside and other flavonoids equal to 0.18 ± 0.05 mg VRE·day^−1^ in EX groups and 0.35 mg VRE·day^−1^ in SL groups. The results demonstrated that the high-fat diet used in the experimental protocol was able to induce obesity (29.38 ± 2.78 g final weight gain), compared to the CT group (15.16 ± 1.27 g final weight gain) (*p* < 0.001) and induced metabolic disorders. HFD-fed mice demonstrated increased fasting glucose (CT group: 232.4 ± 15.93 mg·dL^−1^ and HF group: 296.75 ± 58.24 mg·dL^−1^, *p* = 0.0386) and total serum cholesterol levels after eight weeks (CT group: 270.79 ± 54.72 mg·dL^−1^ and HF group: 347.02 ± 21.34 mg·dL^−1^ (*p* = 0.0378). Dried stalks and leaves promoted a reduction (*p* = 0.0386) in fasting blood glucose (HFSL group: 208.75 ± 41.56 mg·dL^−1^), compared to the HF group (296.75 ± 58.24 mg·dL^−1^); however, the beet stalks and leaf extract did not improve the fasting blood glucose level (HFEX group: 244.5 ± 26.21 mg·dL^−1^), compared to HF group (Figure 4).

The addition of the beet stalks and leaf extract (*p* = 0.0171) or dehydrated beet stalks and leaves (*p* = 0.0254) was able to improve total cholesterol levels (HFEX: 309.14 ± 11.07 mg·dL^−1^ and HFSL: 294.72 ± 34.20 mg·dL^−1^), compared to the HF group (347.02 ± 21.34 mg·dL^−1^) (Figure 4). Despite the favorable effects of the use of the dehydrated beet stalks and leaves in the high fat diet (HF groups), supplementation of the control diet (CT groups) with dehydrated beet stalks and leaves or the ethanolic extract of beet stalks and leaves did not exert any effect on weight gain, blood glucose levels and total cholesterol levels in mice.

### 3.4. The Effect of Beet Stalks and Leaves on Oxidative Stress and Antioxidant Enzyme Activities

**Figure 5:**
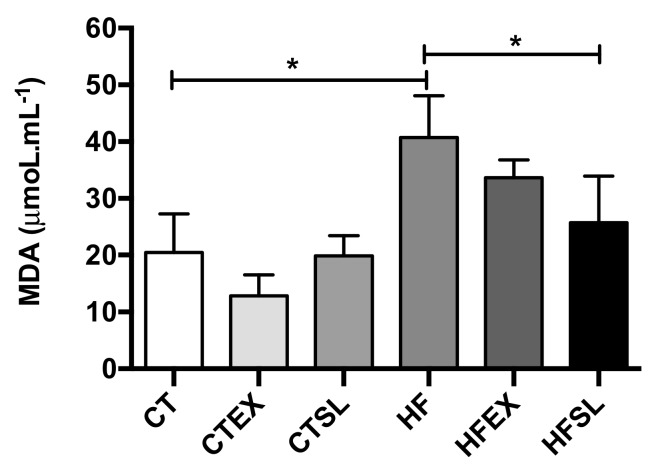
Hepatic MDA concentrations (μmoL·mL^−1^) at the end of the experiment (*n* = 5 animals per group). CT: control group; CTEX: control group supplemented with beet stalks and leaves ethanolic extract; CTSL: control group supplemented with dehydrated beet stalks and leaves; HF: high-fat diet; HFEX: high-fat diet supplemented with beet stalks and leaves ethanolic extract; HFSL: high-fat diet supplemented with stalks and dried beet leaves. * Statistically significant difference (*p* < 0.05).

**Figure 6:**
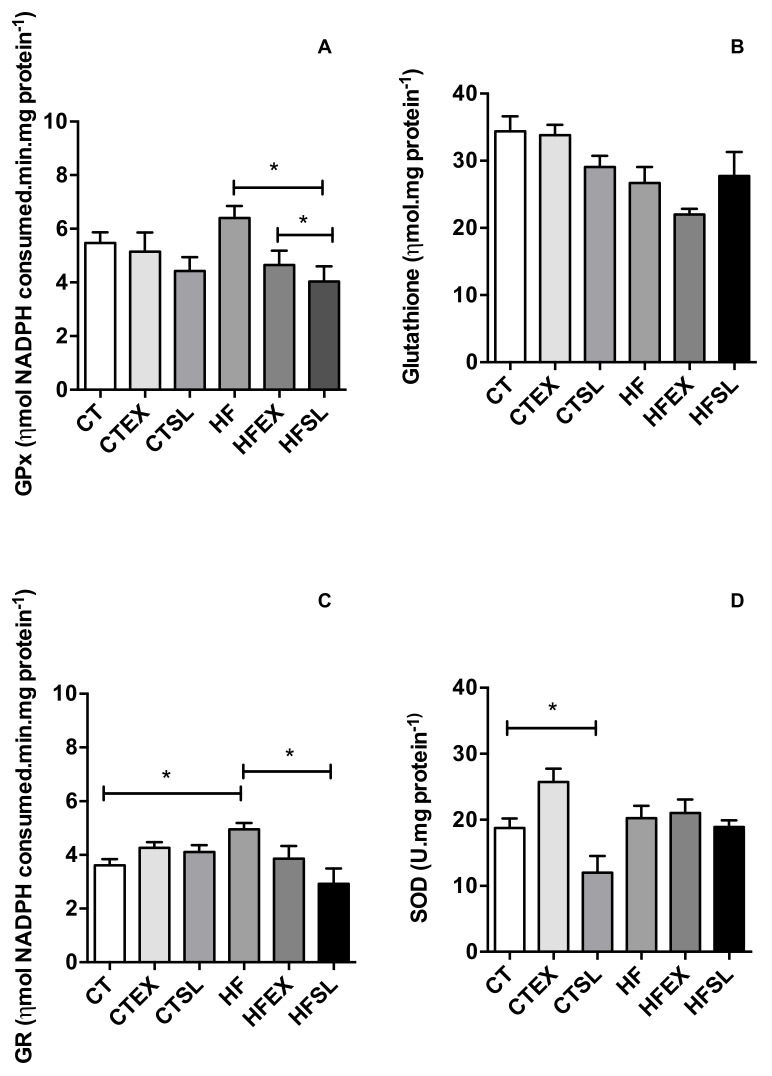
Activity of the hepatic antioxidant enzymes. (**A**) superoxide dismutase (SOD); (**B**) total glutathione (GSH); (**C**) glutathione peroxidase (GPx); (**D**) glutathione reductase (GR) at the end of the experiment (*n* = 5 animals per group). CT: control group; CTEX: control group supplemented with beet stalks and leaves ethanolic extract; CTSL: control group supplemented with dehydrated beet stalks and leaves; HF: high-fat diet; HFEX: high-fat diet supplemented with beet stalks and leaves ethanolic extract; HFSL: high-fat diet supplemented with stalks and dried beet leaves. * Statistically significant difference (*p* < 0.05).

The HF diet efficiently induced oxidative stress in animals after eight weeks, as demonstrated by an increase in hepatic MDA levels (Figure 5). However, supplementation of the HF diet with dehydrated beet stalks and leaves decreased (*p* = 0.0174) MDA levels (HFSL group: 25.71 ± 8.17 μM·mL^−1^ and HF group: 40.71 ± 5.46 μM·mL^−1^), in association with reductions in GPx activity (HF group: 6.40 ± 0.78 ηmol NADPH consumed.min.mg ptn^−1^ and HFSL group: 4.04 ± 0.98 ηmol NADPH consumed·min·mg·ptn^−1^) (*p* = 0.0171) and GR activity (HF group: 4.95 ± 0.37 ηmol NADPH consumed.min.mg·ptn^−1^and HFSL group: 2.91 ± 1.00 ηmol NADPH consumed·min·mg·ptn^−1^) (*p* = 0.0169) as shown in Figure 6. Supplementation of the HF diet with beet stalks and leaves did not change SOD activity levels (HF group: 20.23 ± 3.22 mg·ptn^−1^ and HFSL group: 18.91 ± 2.06 mg·ptn^−1^) (*p* > 0.05). However, SOD activity was the only parameter that presented a reduction in the CT group supplemented with dehydrated beet stalks and leaves.

## 4. Discussion

Similar to other studies, the high-fat diet was able to induce abdominal obesity, impaired glucose tolerance and dyslipidemia after 8 weeks [34,35]. Supplementation with dehydrated beet stalks and leaves prevented many of the alterations that resulted from obesity in mice, decreasing fasting glucose and cholesterol levels. These results may be associated with the intake of Vitexin-2-*O*-rhamnoside-related compounds, as demonstrated in previous studies [18,21,36,37]. The flavonoid glycosides derived from apigenin, denominated Vitexin (i.e., Vitexin-2-*O*-rhamnoside and Vitexin-2-*O*-xyloside), could be responsible for the inhibition of α-glucosidase activity [19,20] and consequently mediate this hypoglycemic effect [38]. Moreover, β-aldehydes and phenolic compounds found in beetroot [21] as well as in beet stalks [18] have been linked to increased low-density lipoprotein (LDL) resistance to oxidation and to the prevention of cardiovascular diseases by reducing the oxidative effects of free radicals on lipids [21,23]. In the study conducted by Tassel et al. (2010), the authors observed high levels of flavonoids in hawthorn leaves (*Crataegus* spp.), such as vitexin, hyperoside, rutin and Vitexin-2″′-*O*-*α*-l-rhamnoside [22]. The authors suggested these phytochemical compounds might be associated with the beneficial effects of this plant on the cardiovascular system as they are able to reduce the ratio between LDL cholesterol (LDL-C) and serum cholesterol (TC) levels [22,36,37].

The induction of obesity by the high-fat diet was able to induce oxidative stress in mice, as demonstrated by high MDA levels and GR activity in the HF group, compared to the CT group. The excess of lipids can contribute to increase free radical production, in turn activating the transcription factor-erythroid 2-related factor 2 (Nrf 2) and increasing the expression of endogenous antioxidant enzymes [39]. This pathway may explain the increase in GR activity in the HF group. However, the increase in GR activity was not enough to prevent the damage from reactive species, as demonstrated by the increase in MDA levels in the HF group. Interestingly, flavonoid glycosides, derived from apigenin, present in beet stalks and leaves may have acted as natural exogenous antioxidants, reducing or minimizing the oxidative stress in mice that were fed on a high-fat diet, similarly to the observations of other studies [40,41,42]. This effect may be due to the antioxidant activity of polyphenols present in fruits and vegetables, which may reduce the requirement for antioxidant enzymatic function by up-regulating Nrf 2-dependent antioxidant enzymes [43]. As a consequence, there was no alteration in the activities of antioxidant enzymes, such as GPx [43].

In a previous study, Kujawska et al. (2009) reported that pretreatment with beetroot juice containing different phytochemicals, such as phenols and flavonoids, caused a partial recovery of glutathione peroxidase and glutathione reductase activity after depletion by the CCl4 treatment [16]. Similarly, Fustinoni-Reis et al. (2016) observed that the high concentration of antioxidant compounds in tucum-do-cerrado fruit (*Bactris cetrosa* Mart.) reduced intestinal GPx activity when oxidative stress was induced by an iron-supplemented diet [43]. Thus, when the organism is exposed to any adverse situation that generates a higher production of ROS, potential therapeutic health benefits can be observed as a function of the dose and time of the administration of vegetables containing bioactive compounds such as Vitexin-2-*O*-rhamnoside and Vitexin-2-*O*-xyloside, which are present in beet stalks and leaves [19].

The present study compared the effect of two types of supplementation on CT and on HF diets; namely, an ethanolic extract and the dehydrated powder of beet stalks and leaf. Beet stalks and leaves are considered non-conventional foods and the dehydration process at elevated temperatures (180 °C) was aimed to facilitate the domestic use and storage. On the other hand, concentration of bioactive compounds in the extract can be much higher than those found in in beet stalks and leaves. However, our results indicated that the use of dehydrated leaves was more biologically active than the ethanolic extract. The differences in the biological effects observed between treatments (SL vs. EX) may be related to the interactions of the phenolic compounds with other components of the sample, especially proteins, present in the food matrix. It is known that polyphenols can interact with proteins, leading to the formation of soluble or insoluble complexes, which in turn may affect their absorption and biological activity [44]. Several aspects will influence protein-polyphenol interactions, such as hydrophobicity, the presence/absence of some functional groups, steric hindrance and spatial arrangements [45]. Enhanced polyphenol-protein interactions are associated with one or more hydroxyl groups in the B-ring (e.g., 3′,4′ dihydroxylated B ring catechol group) of flavonoids and the presence of an unsaturated 2,3-bond in conjugation with a 4-carbonyl group, which are characteristics observed in the structure of vitexin (Figure 3) [46]. These types of interactions can both increase and decrease the biological activity of phenolic compounds due to the formation of protein-polyphenol complexes and can also cause an increase in the antioxidant activities of the protein molecules. For example, there are reports of synergistic effects of phenolic compounds with acidic di and tripeptides, such as glu-glut and asp-asp-asp, on plaque aggregation, increasing their potential protective effects against atherosclerosis [46,47]. Thus, the synergism between the proteins present in the SL groups and flavonoids may contribute to the additive effect observed in these groups, when compared to the groups that only received the ethanolic extract, suggesting the existence of protein-flavonoid interactions that increase the biological effect.

Furthermore, binding of polyphenols affects the secondary and tertiary structures of protein molecules and may affect the activity of digestive enzymes [44], affecting the absorption of nutrients by the SL fed groups. As such, the route of administration of the extract should be further investigated, focusing on the interaction of the phenolic compounds present in the extract with other components of the diet, especially proteins, and the duration of the intervention, in order to understand the underlying mechanism involved and how to maximize their potential benefits in the organism.

## 5. Conclusions

The use of non-conventional foods such as beet stalks and leaves, in addition to reducing food waste, can help reduce the liver damage caused by a high-fat diet and improve the alterations in metabolic parameters due to the presence of flavonoids, such as Vitexins. Furthermore, based on the present findings, we intend to evaluate the recommended dose and the appropriate route of administration of these compounds for a better understanding of the metabolic pathways involved, and assess the effect of beet stalks and leaves on the gut microbiota.

## Figures and Tables

**Table 1:** Chemical compositions of the control and high-fat diets used in this study (g·100 g^−1^).

	Groups	
Nutrients (g·100 g^−1^)	Control Diet	High-Fat Diet
Proteins	11.02	15.68
Lipids	4.28	34.34
Carbohydrates	70.09	33.93
Energy (kcal)	362.78	507.50

**Table 2:** Ingredient compositions of the control and high-fat diets (g·100g^−1^).

Ingredients	Standard			60% High-Fat		
	Standard (CT)	Stalk and Leaf Extract (CTEX)	Dried Stalks and Leaves (CTSL)	Saturated Lipids (g) (HF)	Stalk and Leaf Extract (HFEX)	Dried Stalks and Leaves (HFSL)
**Starch**	41.07	41.07	41.07	2.00	2.00	2.00
**Casein**	11.00	11.00	11.00	16.10	16.10	16.10
**Wheat flour**	20.00	20.00	20.00	24.00	24.00	24.00
**Ethanolic extract of beet stalks and leaves**	--	19.00 mL	--	--	19.00 mL	--
**Dehydrated beet stalks and leaves**	--	--	0.50	--	--	0.50
**Dextrinized corn starch**	11.50	11.50	11.50	3.91	3.91	3.91
**Sucrose**	2.50	2.50	2.50	10.00	10.00	10.00
**Soybean oil**	4.00	4.00	4.00	4.00	4.00	4.00
**Lard**	-	-	-	30.00	30.00	30.00
**Cellulose microfiber (fiber)**	5.00	5.00	4.50	5.00	5.00	4.50
**Mineral mix**	3.50	3.50	3.50	3.50	3.50	3.50
**Vitamin mix**	1.00	1.00	1.00	1.00	1.00	1.00
**L-cystine**	0.18	0.18	0.18	0.24	0.24	0.24
**Choline bitartrate**	0.25	0.25	0.25	0.25	0.25	0.25
**Total**	100	100	100	100	100	100

Footnotes: CT: standard diet; CTEX: standard diet with stalk and leaf extract (190 mL of extract); CTSL: standard diet with dried stalks and leaf (0.5% of dried stalks and leaves).; HF: high-fat diet (60% lipids); HFEX: high-fat diet with stalk and leaf extract (60% lipids and 19 mL of ethanolic extract); HFSL: high-fat diet with dried stalks and leaf (60% lipids and 0.5% dehydrated beet stalks and leaves).

**Table 3:** Chemical composition (mean ± SD) of dehydrated beet stalks and leaves (*Beta vulgaris* L.), expressed as g·100 g^−1^ stalks and leaves (dry basis).

Nutrients	g·100 g^−1^
Moisture	31.62 ± 3.39
Ashes	11.11 ± 4.07
Dietary Fiber	0.34 ± 0.05
Proteins	0.59 ± 0.08
Lipids	0.2 ± 0.00
Carbohydrates *	56.14

* Total carbohydrates were calculated by the difference.

**Table 4:** Ions detected (*m*/*z*) in the extracts. * indicates a major peak in the chromatogram. The numbers in bold indicate compounds showing the ion (*m*/*z*) 293.

Peak Number	Retention Time (min)	Ions (M-H)-(*m*/*z*)	Remarks
1	1.28–1.53	215, 242	
2	1.53–1.76	215, 242, 377, 404	
3	2.0–2.2	117, 183, 232	
4	2.9–3.1	289	
5	9.2–9.4	312, 328	
6	15.0–15.45	210, 323, 355, 387, 487, 1087	
7	18.8–19.1	275, 405, 433, 563, 593	
8	19.1–19.35	433, 563, 593, 639	Mass 563, 593: Frag. M/Z 293 detected.
9 *	19.5–20	563, 577, 1127	Mass 563, 577, 1127: Frag. M/Z 293 detected.
10	20.5–20.7	635	Mass 635: Frag. M/Z 293 detected
11	20.7–21.0	605	Mass 605: Frag. M/Z 293 detected
12	21.1–21.3	679	Mass 679: Frag. M/Z 293 detected
13	21.3–21.45	609,679	Mass 609,679: Frag. M/Z 293 detected
14 *	21.7–22.2	605	Mass 649: Frag. M/Z 293 detected
15	22.3–22.5	342, 605, 649	Mass 605, 649: Frag. M/Z 293 detected
16	23.3–23.56	405 ,451, 577	
17	23.6–23.7	635, 713, 723, 740	Mass 635: Frag. M/Z 293 detected
18 *	23.96–24.24	605, 641, 651, 668	Mass 605, 641, 651: Frag. M/Z 293 detected
19	24.26–24.54	307, 605, 836	Mass 605: Frag. M/Z 293 detected
20	25.8–26	619, 361, 883	
